# Non-Covalent Isotope Effects

**DOI:** 10.1021/acs.jpclett.3c00610

**Published:** 2023-04-12

**Authors:** Mateusz Pokora, Agata Paneth, Piotr Paneth

**Affiliations:** † International Center of Research on Innovative Biobased Materials (ICRI-BioM) − International Research Agenda, Lodz University of Technology, Stefanowskiego 2/22, 90-924 Lodz, Poland; ‡ Chair and Department of Organic Chemistry, Faculty of Pharmacy, Medical University of Lublin, Chodzki 4A, 20-093 Lublin, Poland; § Institute of Applied Radiation Chemistry, Lodz University of Technology, Zeromskiego 116, 90-537 Lodz, Poland

## Abstract

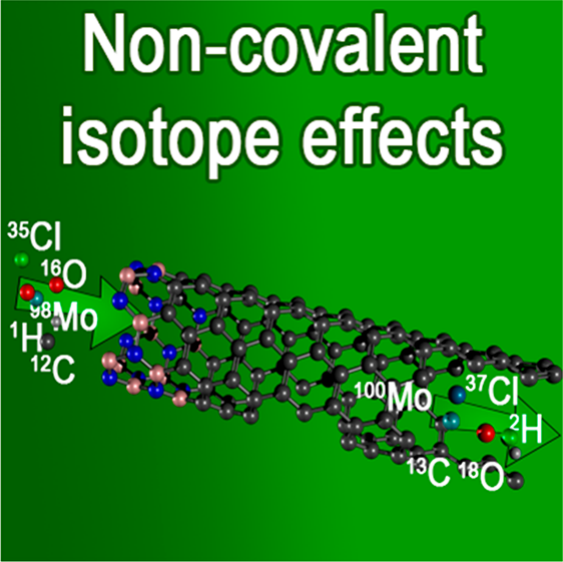

In this Perspective, we present examples of isotope effects that originate from noncovalent interactions, mainly hydrogen bonding, electrostatics, and confinement. They are traditionally widely used in isotopic enrichment processes, as well as in studies of mechanisms of different (bio)chemical and physical phenomena. We then show the emerging areas of their applications, mainly medical and material sciences. We stress that these emerging applications require either high enrichment or isotopic substitution, which requires the development of new effective techniques of isotopic purification.

The history of isotope effects^1^ is strongly coupled with the need for isotopically enhanced materials, which can be achieved in several different physical processes. In most of them, noncovalent interactions play the main role in changes of isotopic composition between initial and final states, called isotopic fractionation. A mathematical description of isotope effects (IE)^2^ derived from statistical thermodynamics in simple cases of thermodynamic equilibrium processes has the form:
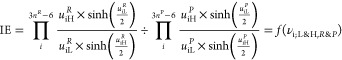
1where *n* is the number of vibrational degrees of freedom (of the initial state *R* and final state *P*), L and H stand for light and heavy isotopic species, and *u*_i_ = *hν*_i_/(*k*_B_*T*), where ν_i_ is the isotopic frequencies of normal modes of vibrations, *T* is absolute temperature, and *h* and *k*_B_ are Planck’s and Boltzmann’s constants, respectively.

As illustrated by the last term in this equation, these isotope effects are directly related to the sets of isotopic frequencies of both states. Thus, isotope effects can be related to changes in the stiffness of the environment around the isotopic atom, i.e., either the force constants of bonds to the isotopic atom or their number differs between the *P* and *R* states. In the case of noncovalent isotope effects, the changes in force constants are usually small, and the interactions with the environment are the main source of isotope effects.

Noncovalent isotope effects can also manifest themselves in kinetic processes when an isotopic atom is not directly involved in a bond-making and/or bond-breaking event and, thus, its vibrations are not coupled with the (imaginary) frequency of crossing the energetic barrier (so-called secondary kinetic isotope effects). Consider for example a simple S_N_2 reaction between chlorine anion and bromomethane, whose reactants, transition state, and products are shown in Figure 1. Although hydrogen atoms are spectators in the process of forming the Cl–C bond and breaking the C–Br bond, the deuterium isotope effect is strongly inverse in the case of D_3_ substitution.^3,4^ This is because hydrogen atoms in the transition state are in a more rigid environment; there are more neighboring atoms compared with the initial state, they are forced into one plane, and the C–H bond lengths become shorter (their force constants increase).

**Figure 1:**
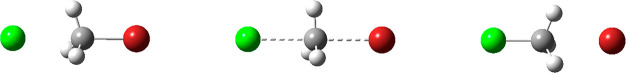
Reactants (left), transition state (middle), and products (right) of an S_N_2 reaction.

The major noncovalent interactions that influence isotopic fractionation include electrostatics, hydrophobic effects, π-stacking, metal coordination, and hydrogen bonding.^5^ They manifest themselves in a wide number of physical and (bio)chemical processes, such as adsorption, chromatographic separation, phase equilibria, (bio)chemical equilibria, and binding of ligands to receptors, to name the most important ones. In this Perspective, we first present examples of “traditional” noncovalent isotope effects and subsequently discuss their expected developments and new possible applications in studies and practice. This contribution is not intended to provide exhaustive literature coverage but rather to present each case in the light of recent examples (where available) with preference given to our work wherever possible.

One of the strongest noncovalent interactions is hydrogen bonding. Hydrogen bonds are also the most frequently present bond in many chemical, and especially biochemical, systems. They manifest themselves in the binding of ligands to receptors [e.g., in biochemistry binding of inhibitors (drugs) to enzymes] and in phase transfer phenomena, such as vapor pressure or chromatography, to name a few.

As an illustration of isotope effects on binding, we use the example of oxamate binding to lactate dehydrogenase (LDH), which was the first experimental documentation of a significant isotope effect on binding that had previously been neglected in the analysis of isotope effects on enzymatic reactions. It has been shown that the oxygen binding isotope effect of two carboxyl oxygen atoms of oxamate exhibits an inverse isotope effect of 0.98.^6^ This strongly inverse (less than unity) value of the heavy-atom (oxygen) isotope effect has been ascribed to bifurcated hydrogen bonding between the carboxyl group of oxamate and the amino acids of the LDH binding site (see Figure 2) on the basis of the density functional theory (DFT) calculations.^7,8^

**Figure 2:**
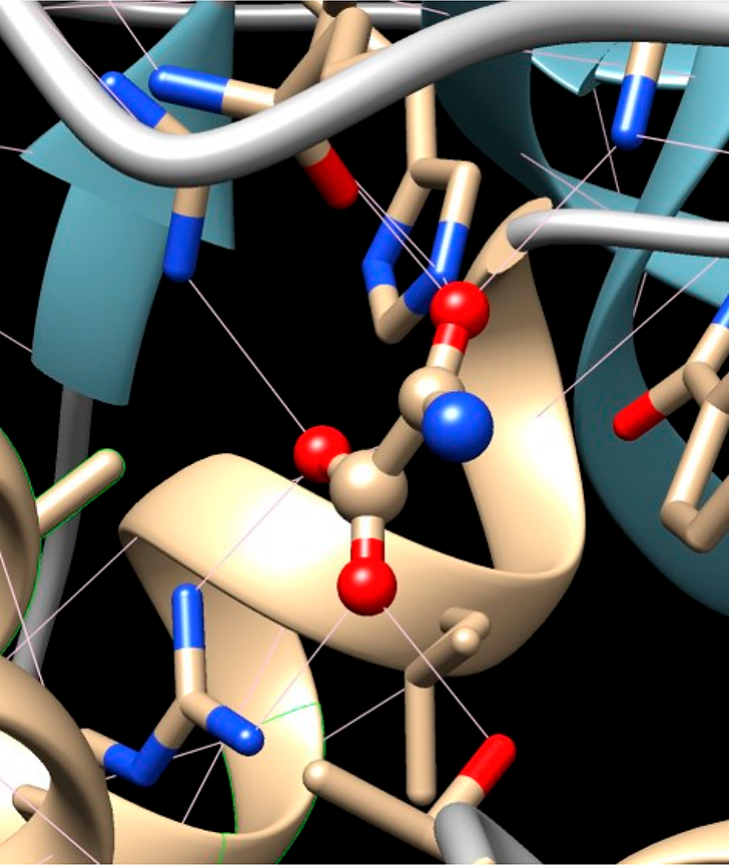
Hydrogen bonding of oxamate (rendered as balls and sticks) with two arginines and threonine of the LDH active site. Carbon atoms are shown in light brown, oxygen atoms are in red, and nitrogen atoms are in blue.

The oxamate–LDH example illustrates the coherence of experimental and computational techniques in studying the details of ligand–receptor interactions. We have also shown that binding isotope effects can be used to identify the binding site in the case of HIV-1 reverse transcriptase.^9^ They are also important in the analysis of kinetic isotope effects on enzymatic reactions since they can contribute to the overall value^10^ in a way that may alter mechanistic interpretations.

Phase transfer equilibria and associated isotope effects^11,12^ are vast areas that can only briefly be touched on here. They play an important role in a plethora of different disciplines, including geochemistry, food authentication, and of course isotopic separation techniques. Only a few selected cases will be presented here to illustrate problems and possibilities.

We start the discussion with an example of bridging isotope effects on phase equilibria and hydrogen bonding: the vapor pressure isotope effect on the vaporization of water. We concentrate here on the bulk result, which avoids a complicated analysis of interfacial dynamics.^13^ The experimental value for the ^18^O isotope effect is 1.0091 ± 0.0002.^12^ Simple modeling of this process by assuming a dielectric continuum model of bulk water for the condensed phase (the model illustrated by the left panel of Figure 3) and the isolated water molecule for the gas phase yields the inverse value of 0.9957. When, instead of the continuum model, explicit hydrogen bonds are considered (illustrated by the right panel of Figure 3), the result of 1.0081 is in excellent agreement with the experiment.^14^

**Figure 3:**
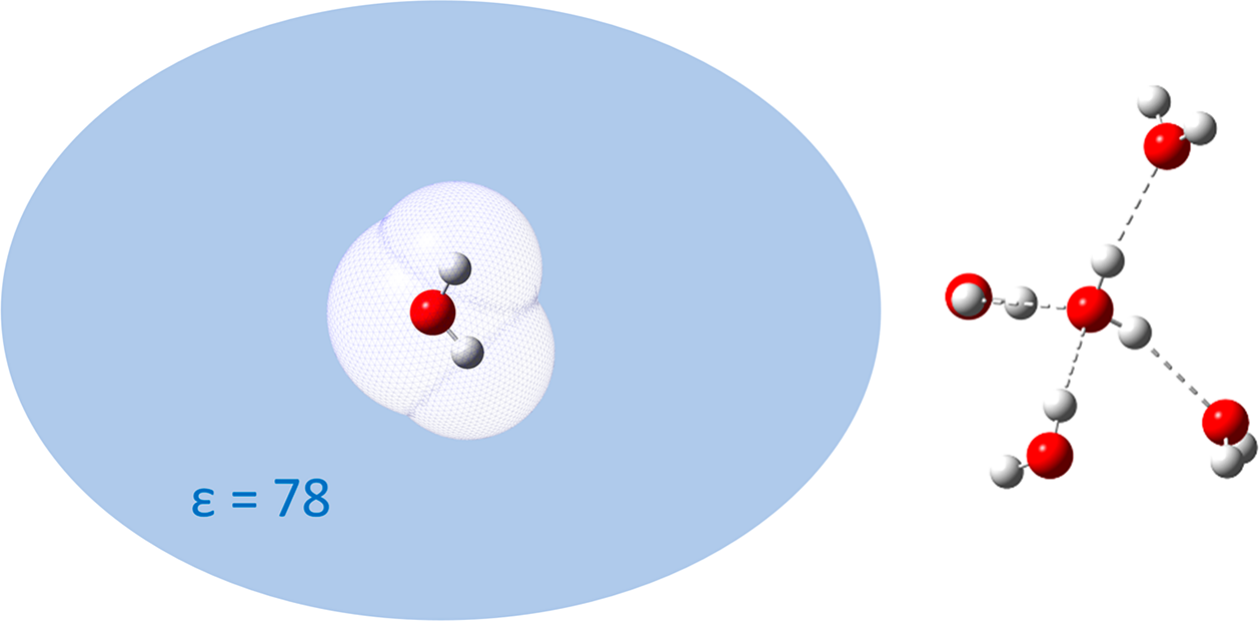
Models of a water molecule in the condensed phase. Left: in a dielectric continuum. Right: in a hydrogen-bonding network. Hydrogen atoms are rendered in white, and oxygen atoms are in red.

A simple explanation for the normal (greater than unity) value of the isotope effect described above is that molecules with heavier isotopes are expected to undergo the evaporation process more slowly, thereby resulting in the enrichment of the condensed phase in the heavier isotope. This intuitive interpretation, however, may fail in the case of more complex molecules,^15^ and a transition between normal and inverse isotope effect occurs at certain temperatures depending on the balance between contributions from the intermolecular and zero-point energy degrees of freedom.^16−18^ As early as 1961, Bigeleisen emphasized the crucial role of molecular structure in the phenomenon.^15^ Many organic compounds (for example, acetic acid,^19^ benzene^20−22^ and its derivatives,^23,24^ and hydrocarbons^21,22,25,26^), as well as simple molecules at higher temperatures, show inverse isotope effects for elements such as hydrogen and carbon, i.e., the condensed phase becomes depleted in the heavier isotope. This is often explained by weaker binding energies that originate mainly from van der Waals interactions in molecules containing heavier isotopes, which results in higher vapor pressure for the heavier isotopologues.^27,28^ The direction of the isotope effect in these processes is sometimes difficult to predict.^29,30^

As an example of the breadth of noncovalent isotope effects applications, it is worth noting that the HDO/H_2_O abundance ratio is used to examine the Martian atmosphere to investigate the early day water reservoir on Mars and how it has evolved with time.^31,32^ As HDO is present both in the atmosphere and on the surface as ice, the ratio of HDO/H_2_O in the gas phase and the ice can be used as a tracer of the aqueous history on Mars.^33^ Isotope fractionation of heavy atoms, such as zinc or chromium, during condensation and evaporation can also be used as a time machine to trace the process of a planet’s formation.^34^

Isotope effects have also been observed in different types of chromatographic techniques. From the historical perspective, it is worth starting with the isotopic separation of HD and D_2_ by gas chromatography.^35^ This technique continues to be used for the analysis of deuterated compounds.^36^ In light of current battery technology, it is also worth mentioning an early work on the enrichment of isotopes by ion exchange chromatography that has been successfully achieved for lithium isotopes by chemical exchange with zeolites.^37^

One of the most precise methods of determining isotopic composition is isotope ratio mass spectrometry (IRMS). However, a sample is usually initially combusted to a gaseous form, e.g., carbon dioxide for the measurements of carbon isotopic composition. This procedure inevitably leads to bulk isotopic composition. The emerging alternative that allows for position-specific isotopic analysis is the use of nuclear magnetic resonance spectroscopy (NMR) for the study of isotopic contents. This technique is already implemented in, for example, food authentication, where variations in the natural abundance isotopic contents of deuterium at methyl and methylene groups are determined (SNIF-MNR technique). Analogous techniques for the isotopic contents of other elements are being developed. They have recently been used in studies of position-specific isotopic analysis of the isotopic composition of paracetamol during chromatographic analysis.^38^ These studies indicated that isotopic fractionation is not only different at different positions of an atom in the molecule but also depends on interactions with the type of the stationary phase; for the six carbon atoms, variation in position-specific isotopic fractionation was higher than 1.0033, while nitrogen isotopic fractionation differed by 1.0046 between cellulose and silica gel.

Applications of the isotope effects in the field of metal complexes are mostly associated with organometallic chemistry, and reviews on kinetic isotope effects are available in the literature.^39,40^ Newer studies suggest that inflamed areas of the body contribute to changes in ^65^Cu content,^41−43^ which could be used for prognosis in end-stage cancer. It is, however, unclear if the observed variation in the isotopic composition is of noncovalent origin. In this respect, reports on the change of isotopic composition of zinc complexes with the coordination number (the higher the number is, the more enriched in the lighter isotope the complex becomes) seem to be more in line with the isotope effects considered herein.^44,45^ Nevertheless, it is not clear what the relation is between ^66^Zn contents and the binding of zinc to proteins.

The miscibility isotope effects appear to be a highly specialized field with no to little interest paid it thus far. The effects were mostly studied for liquid–liquid systems (a typical example might be the studies by Szydłowski and co-workers^46^ who examined the impact of deuteration and ^18^O/^16^O substitution in the isobutyric acid/water system). The situation, however, may change in the future since miscibility isotope effects have also been identified in solid polymeric systems. This opens great application possibilities in the field of the design of drug delivery systems, capsule shells, and controlled-release tablets.^47^

The earliest attempts to explain the phenomenon on the basis of molecular interactions^48^ were later expanded by Szydłowski and co-workers^49,50^ by taking into account inter- and intramolecular interactions. They emphasized a strong contribution to isotope effects of dipole–dipole interactions in small molecules with a permanent dipole moment, as well as vibrational couplings.

In the field of polymer chemistry, a lot of studies regarding polymer blends utilizing the deuteration of polymers to influence their miscibility can be found, but recently the miscibility isotope effect has been used not only to facilitate the mixing process of two polymers but also to tune the final properties of the blend,^51,52^ as the miscibility of polymers governs the mechanical properties of the blend.

To study the influence of the electrostatics on the isotope effects, we have carried out theoretical (DFT, ωB97x-D/def2-TZVP) calculations on the influence of interacting with ions (Na^+^, Li^+^, F^–^, Cl^–^, and Br^–^), neutrals (He, Ne, Ar, and Xe), and continuum models of the environment (dielectric properties of the Ar matrix, water, and formamide) on the stretching vibration of hydroxyl anion. Subsequently, we have evaluated isotope effects on a putative transition of isolated hydroxyl to an environment in which either other ions/neutrals are present or the bulk dielectric constant is different. The results are illustrated in Figure 4. Interestingly, a good correlation between the deuterium isotope effect and O–H bond length is obtained for both optimized and even unoptimized (orange-labeled points in Figure 4) structures. This is not the case with the ^18^O isotope effect where such correlation can be found only for the converged geometries. The lesson to be taken from these calculations is that the change in the electrostatic properties of the environment can result in very large isotope effects, especially in the case of hydrogen isotope effects.

**Figure 4:**
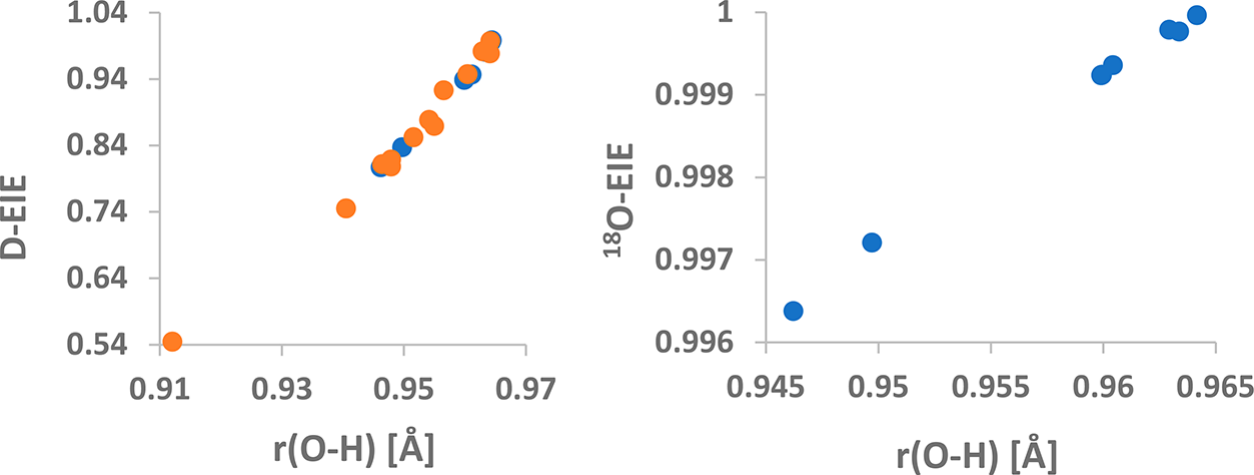
Dependence of the deuterium (left) and oxygen (right) equilibrium isotope effects on the length of the O–H bond (Å) under different stimuli (see text). Points marked by orange color indicate calculations that did not reach full optimization, and blue points indicate fully converged calculations.

Confinement can play a significant role in isotopic fractionation. Using DFT, we have shown^53^ that π–π interactions between benzene adsorbed on graphene (1D confinement) lead to both ^13^C and ^2^H isotope effects, which are orientation-dependent. For the energetically favored orientation (rightmost in Figure 5), the corresponding equilibrium isotope effects for fully isotopically substituted isotopologues were calculated to be 0.998 and 1.017, respectively. Furthermore, calculations indicate that the three orientations can be distinguished on the basis of the isotopic fractionations of carbon and hydrogen.

**Figure 5:**
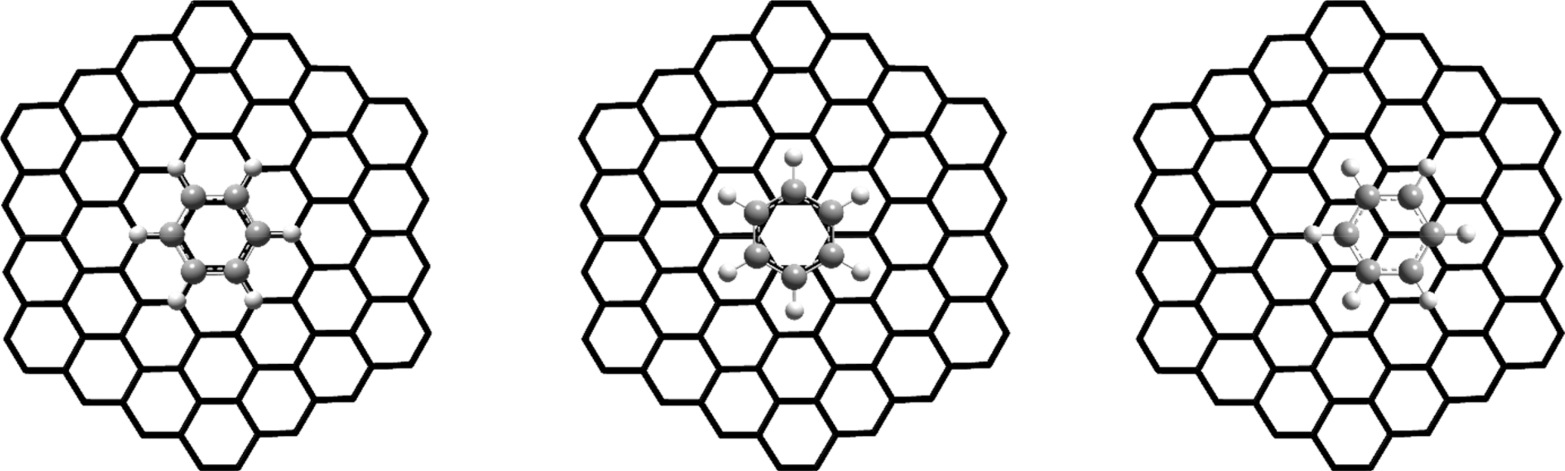
Different orientations of benzene over a graphene waffle (black wire representation). Carbon atoms of benzene are shown in gray, and hydrogen atoms are in white.

2D confinement, as shown in the left panel of Figure 6, of a chlorine ion in a carbon nanotube or full 3D confinement of the same ion in a boron nitride cage also yields substantial isotopic fractionation that is associated with constraints on the vibrations along the principal axes. For the cases shown in Figure 6, significant chlorine equilibrium isotope effects of 0.9715 and 0.9861, respectively, have been calculated.^54^ These values exceed the typical magnitude of kinetic isotope effects.

**Figure 6:**
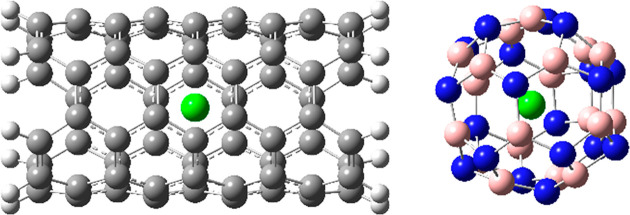
2D and 3D confinement of chlorine anion. Carbon atoms are shown in gray, nitrogen atoms are in blue, chlorine atoms are in green, boron atoms are in pink, and hydrogen atoms are in white.

While we expect that the traditional use of noncovalent isotope effects will continue to play an important role in isotope separation techniques^1^ and studies of the mechanisms of chemical and biochemical^55^ reactions, the most exciting progress seems to be ahead of us in their medical and nanotechnological applications. Below, we exemplify a few recent cases.

The emerging venue of medical applications of isotopic materials takes advantage of the difference in decomposition time of isotopically modified drugs (at present only deuteration is considered). Pharmacologically, the noncovalent isotope effect can result in different metabolic rates, and thus, the half-life of the isotopic drug may increase. This, in turn, can lead to a reduction of the required drug dose and, in consequence, lower toxic and side effects. The first deuterated drug approved by the FDA in 2017^56^ was AUSTEDO (deutetrabenazine). In this, (3*S*,11*bS*)-3-(2-methylpropyl)-9,10-bis-methoxy-1,3,4,6,7,11*b*-hexahydrobenzo[a]quino-lizin-2-one protium atoms in two methoxy groups are substituted by deuterons, as illustrated in Figure 7. Deuteration doubles the half-life of the active metabolites,^57^ thereby allowing for the reduction of the dose by one-third.

**Figure 7:**
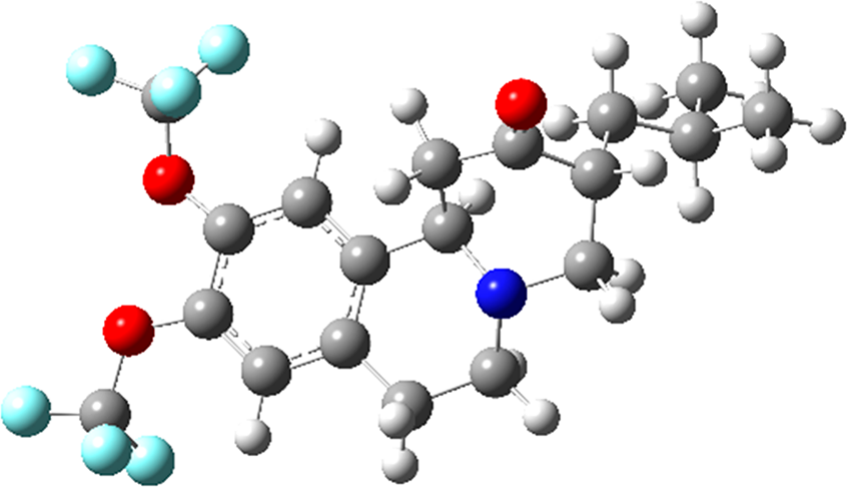
Structure of AUSTEDO with positions of deuteration (in methoxy groups) marked by light blue, while other hydrogen atoms are rendered in white. Carbon atoms are shown in gray, oxygen atoms are in red, and the nitrogen atom is in blue.

More than 20 deuterated drugs are currently being scrutinized for their pharmacokinetic effects, such as efficacy, but also for their safety.^58,59^ To date, drug deuteration has been shown to enhance the bioactivation of drugs while reducing the formation of unwanted metabolites and stabilizing therapeutically desired enantiomers.^58^

As mentioned above, miscibility isotope effects on polymer blends may provide an opportunity to exploit them either to improve the solubility of the drug molecule in water, which was shown to be potentially successful,^60^ or to manipulate the miscibility of amorphous drugs with polymer blends by the means of deuterium substitution. As the interactions between the polymer and the drug molecule can affect crystallization and drug diffusivity, altering the strength of the interactions by isotopic substitution seems to be a great field to be explored.

In materials science, a recent finding points out significantly different properties of isotopically pure materials that may lead to exciting new industrial applications in nano- and microelectronics. Among them, deuterated compounds may find their way into digital display technology. Organic light-emitting diodes (OLEDs) offer numerous advantages over liquid crystal displays (LCDs) and light-emitting diodes (LEDs), such as higher flexibility, brightness, and energy efficiency.^61^ However, they have a limited lifetime. Early studies indicate that the use of deuterated material may increase their lifetime^62^ and luminescence^63,64^ without altering their performance. There are also reports of isotope-induced chirality altering the properties of nanomaterials,^65^ which can lead to changes in their characteristics.

As futuristic as the use of isotope abundance in the prediction of diseases may sound, the world of quantum computing utilizes the isotope effects in an even more surprising fashion. Li and co-workers reported that Co(II) complexes form molecule-based qubits with ON/OFF switching behavior regulated by the H/D isotope effect.^66^ As a result of isotopic substitution, the change in vibrational properties of the molecule affects the spin–phonon coupling, which leads to different relaxation times. The importance of isotopes in quantum computing was emphasized by Plekhanov in 2012.^67^ Perdeuterated solvents were used to reduce the solvent impact on decoherence.^68−70^ Cho and co-workers studied theoretically a hydrogen-based qubit encapsulated in a fullerene.^71^ Fullerene perdeuteration eliminates possible interferences in the nuclear spin measurement.

Isotopic substitution affects numerous properties of 2D solid crystals. Recently, one of these attracted much attention because it can have significant consequences in practical applications. It has been shown that thermal dissipation is isotope-sensitive because of the mass dependence of phonon vibrational frequencies. The first experiment of this kind with ^13^C-enriched graphene showed that at 50% abundance, the thermal conductivity was 2 orders of magnitude higher than natural abundance.^72^ Similar effects have recently been observed for a 2D semiconducting material; the in-plane thermal conductivity of isotopically pure ^100^ MoS_2_ is 50% higher than for the natural mixture of molybdenum.^73^ For electronic devices, which are plagued by overheating problems, isotopic editing to improve heat dissipation seems to be a very attractive venue for development.

For the purposes described above, obtaining isotopically pure materials is the key issue. The dominant isotopic modification is, of course, deuteration, which leads to the largest changes. Deuterated solvents serve as the source of deuterium in the synthesis of next generation OLEDs. The separation of H_2_ and D_2_ on metal organic frameworks (MOFs) has been recently gaining both theoretical and experimental attention.^74−77^ Although a plethora of applications of perdeuterated materials emerge in the area of medicine and the design of new materials, in the latter case, the isotopic substitution of heavy atoms may need to develop even faster, as exemplified by the isotopic effect of heat dissipation in the nanomaterial realm. In this area, nontraditional methods of isotopic enrichment seem to be on the way to rapid development. As we have illustrated above, confinement might be one way of achieving increased separation.^53^ While in our studies we have considered carbon nanotubes, their functionalization or change of material to boron nitride,^78^ for example, might prove effective for isotopic enrichment purposes. A similar approach that also may be probably tuned for this purpose is molecular sieving through graphene-based membranes.^79^ Another interesting technique of obtaining an isotopically enriched layer in a particular part of the 2D material for the modification of properties (like thermal conductivity mentioned above) has been proposed recently by Jeong and Seebauer.^80^ They have shown that oxygen diffusion on TiO_2_ can lead to ^18^O enrichment near the surface. This technique should be much more economical than the production of isotopically pure material because it uses isotopic exchange with relatively cheap and available isotopic material rather than tedious isotopic synthesis.

In conclusion, this Perspective shows that although traditional noncovalent isotope effects remain a useful tool for isotopic enrichment and mechanistic studies, it is apparent that new applications in the realm of drug design and material properties are emerging and the latter creates a demand for new enrichment methodologies to be developed.
